# Crystal structure and Hirshfeld surface analysis of *N*-[(*Z*)-(2-hy­droxy­phen­yl)methyl­idene]aniline *N*-oxide

**DOI:** 10.1107/S2056989021004813

**Published:** 2021-05-11

**Authors:** Shaaban K. Mohamed, Awad I. Said, Joel T. Mague, Moustafa F. Aly, Mehmet Akkurt, Sahar M. I. Elgarhy

**Affiliations:** a Chemistry and Environmental Division, Manchester Metropolitan University, Manchester, M1 5GD, England; b Chemistry Department, Faculty of Science, Minia University, 61519 El-Minia, Egypt; c Chemistry Department, Faculty of Science, Assuit University, Egypt; d Department of Chemistry, Tulane University, New Orleans, LA 70118, USA; e Chemistry Department, Faculty of Science, South Valley University, Egypt; f Department of Physics, Faculty of Sciences, Erciyes University, 38039 Kayseri, Turkey; g Faculty of Science, Department of Bio Chemistry, Beni Suef University, Beni Suef, Egypt

**Keywords:** crystal structure, hydrogen bond, N-oxide, C—H⋯π(ring), nitrones, Hirshfeld surface analysis

## Abstract

The conformation of the title compound, C_13_H_11_NO_2_, is partially determined by a strong, intra­molecular O—H⋯O hydrogen bond. The crystal packing consists of strongly corrugated layers parallel to the *ac* plane and associated through C—H⋯π(ring) inter­actions. A Hirshfeld surface analysis of the crystal structure indicates that the most significant contributions to the crystal packing are from H⋯H (44.1%), C⋯H/H⋯C (29.4%) and O⋯H/H⋯O (17.3%) contacts.

## Abstract 2

The conformation of the title compound is partially determined by a strong, intra­molecular O—H⋯O hydrogen bond. In the crystal, C—H⋯O hydrogen bonds link the mol­ecules, forming chains along the *a*-axis direction, which are linked into strongly corrugated sheets parallel to the *ac* plane by C—H⋯O hydrogen bonds and C—H⋯π(ring) inter­actions. The sheets are associated through additional C—H⋯π(ring) inter­actions.

## Chemical context   

Nitro­nes are a very important class of organic compounds as a result of their medicinal and pharmaceutical applications. They show anti­fungal (Salman *et al.*, 2013 ▸), anti­bacterial (Chakraborty *et al.*, 2010 ▸), neuroprotective (Chioua *et al.*, 2012 ▸) and anti­cancer (Floyd *et al.*, 2011 ▸) activities. In addition, nitrone compounds are widely used as anti­oxidant agents (Al-Mowali *et al.*, 2014 ▸) because of their ability to scavenge free radicals. Based on these findings and following our inter­est in this area, we report herein the crystal structure of the title compound.
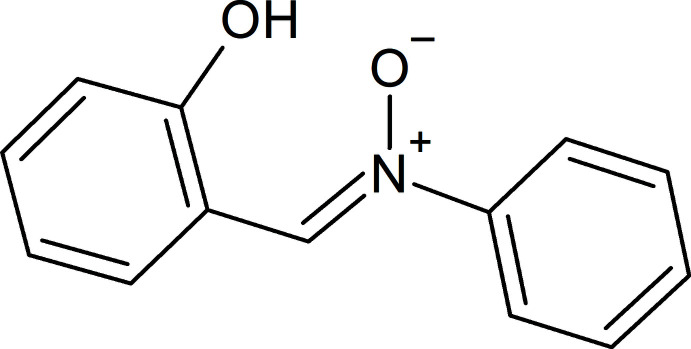


## Structural commentary   

**Figure 1:**
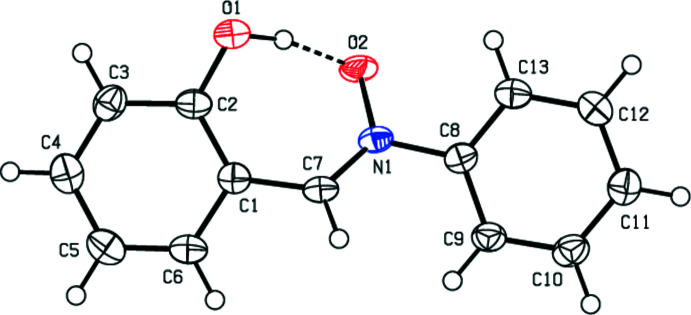
The title mol­ecule with labelling scheme and 50% probability ellipsoids. The intra­molecular hydrogen bond is shown by a dashed line.

The mol­ecular structure of the title compound (Fig. 1 ▸) is almost planar, with maximum deviations of 0.398 (2) Å for O1 and −0.756 (2) Å for O2. The N1—O2 distance of 1.331 (2) Å is normal for a single bond and agrees well with those observed in other amine *N*-oxides. The dihedral angle between the aromatic rings (C1–C6 and C8–C13) is 1.94 (12) °. The torsion angles C2—C1—C7—N1, C1—C7—N1—C8, C1—C7—N1—O2, C7—N1—C8—C9 and O2—N1—C8–C-9 are −30.2 (3), −179.7 (2), −0.4 (3), 27.3 (3) and −152.0 (2)°, respectively. The conformation of the title compound is partially determined by a strong, intra­molecular O1—H1⋯O2 hydrogen bond (Table 1 ▸).

## Supra­molecular features   

**Figure 2:**
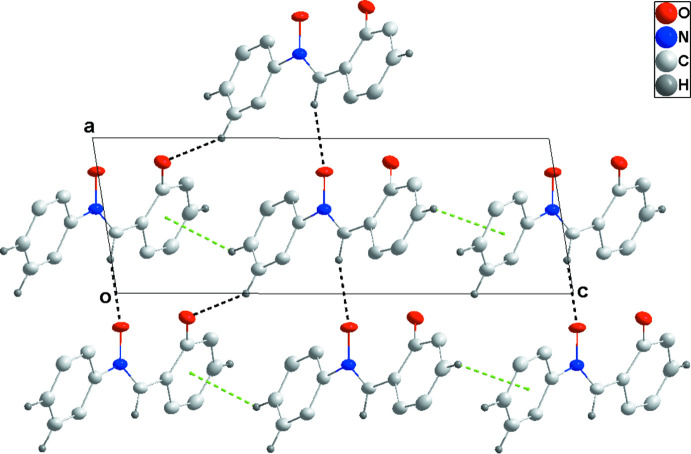
Detail of the inter­molecular C—H⋯O hydrogen bonds and the C—H⋯π(ring) inter­actions (black and green dashed lines, respectively) viewed along the *b*-axis direction.

In the crystal, C7—H7⋯O2^i^ hydrogen bonds (Table 1 ▸) link the mol­ecules, forming chains along the *a*-axis direction. The chains are linked into strongly corrugated sheets parallel to the *ac* plane by C10—H10⋯O2^ii^ hydrogen bonds and C11—H11⋯*Cg*1^iii^ inter­actions (*Cg1* is the centroid of the C1–C6 hy­droxy­phenyl ring; Table 1 ▸ and Fig. 2 ▸). The sheets are stacked along the *b*-axis direction by C4—H4⋯*Cg*2^iv^ inter­actions (*Cg2* is the centroid of the C8–C13 phenyl ring; Table 1 ▸ and Figs. 2 ▸ and 3 ▸).

## Hirshfeld surface analysis   

**Figure 4:**
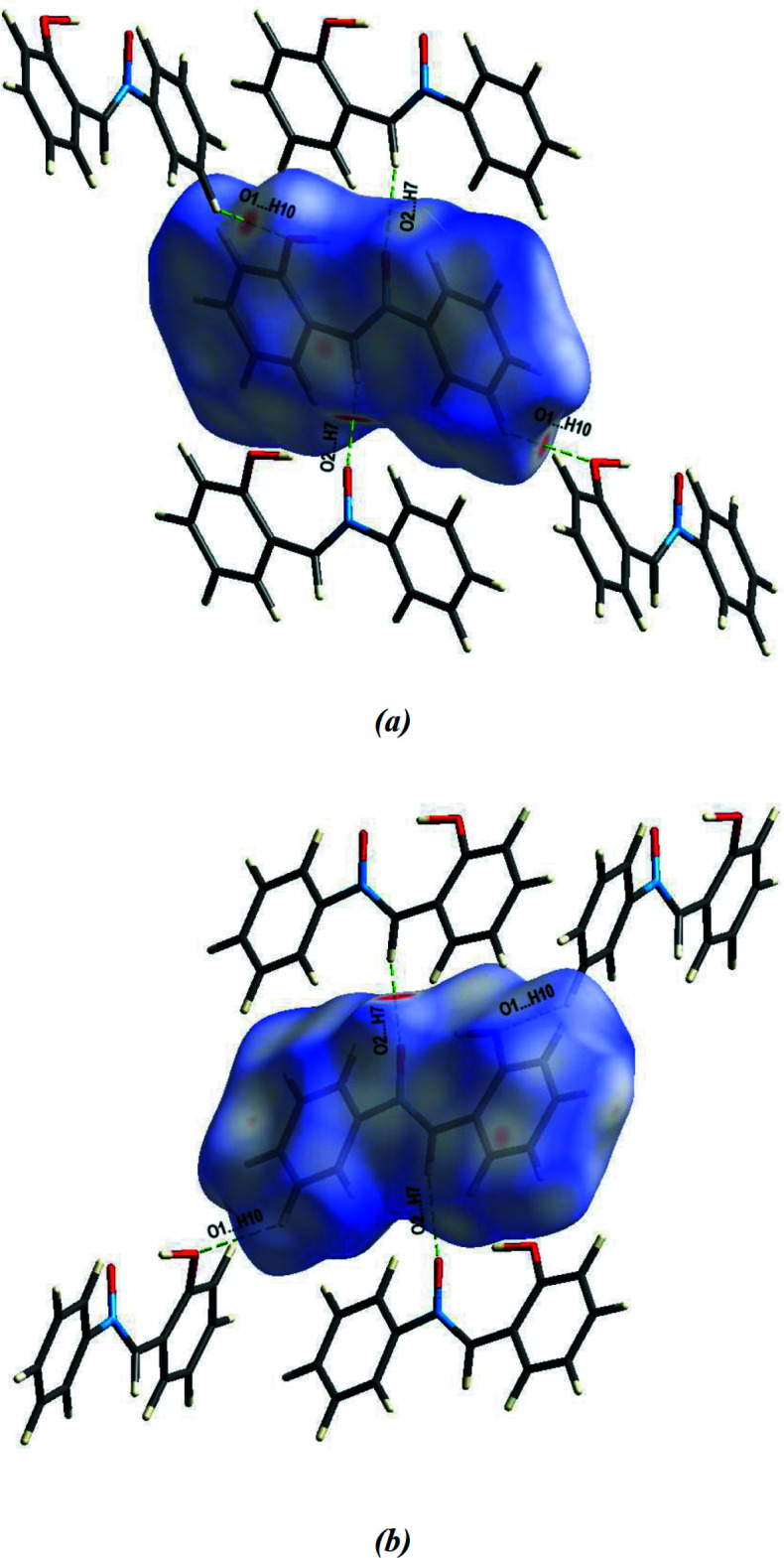
A view of the three-dimensional Hirshfeld surface with the C—H⋯O inter­actions for the title compound, plotted over *d*_norm_ in the range −0.2242 to 1.2146 a.u. (*a*) front view, (*b*) back view.

A Hirshfeld surface analysis (Spackman & Jayatilaka, 2009 ▸) was carried out using *CrystalExplorer17.5* (Turner *et al.*, 2017 ▸) to visualize the inter­molecular inter­actions in the title compound. The Hirshfeld surface mapped over *d*_norm_ (Fig. 4 ▸) shows the expected bright-red spots near atoms O1, O2, H7 and H10, which are involved in the C—H⋯O hydrogen-bonding inter­actions. The bright-red spot near O1 indicates its role as a hydrogen-bond acceptor to (C10)H10 (Fig. 4 ▸) and another red region near O2 correlates with the C7—H7⋯O2 inter­action.

**Figure 5:**
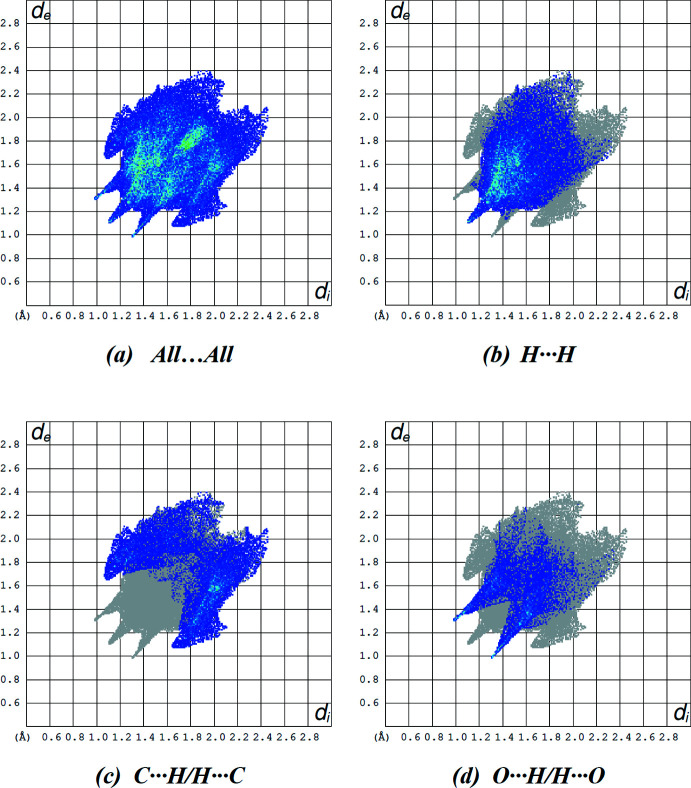
A view of the two-dimensional fingerprint plots for the title compound, showing (*a*) all inter­actions, and delineated into (*b*) H⋯H, (*c*) C⋯H/H⋯C and (*d*) O⋯H/H⋯O inter­actions. The *d*_i_ and *d*_e_ values are the closest inter­nal and external distances (in Å) from given points on the Hirshfeld surface.

The two-dimensional fingerprint plots show the relative contributions of the various types of contacts to the Hirshfeld surface for the title compound (McKinnon *et al.*, 2007 ▸). The plots (Fig. 5 ▸) reveal that H⋯H and C⋯H/H⋯C inter­actions make the greatest contributions to the surface contacts, while O⋯H/H⋯O, C⋯C, N⋯H/H⋯N, N⋯C/C⋯N and O⋯C/C⋯O contacts are less significant (Tables 2 ▸ and 3 ▸).

## Database survey   

The four most closely related structures are (*Z*)-*N*-[(1,3-diphenyl-1*H*-pyrazol-4-yl)methanimine]-*N*-oxido (DEPVOM; Mohamed *et al.*, 2018 ▸), (*Z*)-1,2-bis­(3-bromo­phen­yl)diazene 1-oxide (SIYHAK01; Goswami *et al.*, 2018 ▸), (*Z*)-*N*-benzyl­idene-1-phenyl­methanamine oxide hydrogen peroxide solvate (JELQOJ; Churakov *et al.*, 2017 ▸) and (*Z*)-*N*-(2-chloro­benzyl­idene)aniline *N*-oxide (ERIXEJ; Fu *et al.*, 2011 ▸).

In the crystal of DEPVOM, (101) layers are generated by C—H⋯O hydrogen bonds coupled with C—H⋯π(ring) and offset π–π stacking inter­actions. In the crystal of SIYHAK01, C—H⋯O and C—H⋯Br hydrogen bonds together with offset π–π inter­actions stack the mol­ecules along the *a*-axis direction. In the crystal of JELQOJ, the organic and peroxide mol­ecules are linked through both peroxide O—H donor groups to oxide O-atom acceptors, giving one-dimensional chains extending along the *b-*axis direction. Weak inter­molecular C—H⋯O hydrogen-bonding inter­actions are also present. In the crystal of ERIXEJ, the mol­ecule is stabilized by an intra­molecular C—H⋯O hydrogen bond. The geometry about the C=N bond is *Z* [C—C—N—O torsion angle = −4.2 (3)°] and the phenyl and benzene rings are *trans*-oriented around the C=N bond. The phenyl and benzene rings make a dihedral angle of 56.99 (2)°.

## Synthesis and crystallization   

(*Z*)-(2-Hy­droxy­phen­yl)methyl­idene]benzenimine *N*-oxide (nitrone) was prepared according to the reported procedures (Mobinikhaledi *et al.*, 2005 ▸). 0.7 ml (6 mmol) of salicyaldehyde were added to a warmed solution of 0.8 g (6 mmol) *N*-phenyl­hydroxy­amine in ethanol followed by stirring for 5 minutes, then standing at room temperature in the dark overnight gave the nitrone, which was recrystallized from ethanol in 53% yield; m.p. 387–388 K.

## Refinement   

Crystal and refinement details are presented in Table 4 ▸. The H atom of the OH group was found in difference-Fourier maps, and its positional parameters were fixed using the AFIX 3 instruction in *SHELXL* and were refined with the isotropic displacement parameter *U*_iso_(H) = 1.5*U*_eq_(O). The C-bound H atoms were positioned geometrically, with C—H = 0.95 Å, and constrained to ride on their parent atoms, with*U*_iso_(H) = 1.2*U*_eq_(C). Attempts to determine the absolute structure did not produce a definitive result, *viz*.: Flack *x* = 0.2 (3) by classical fit to all intensities 0.30 (14) from 611 selected quotients (Parsons’ method). A round of TWIN/BASF refinement gave BASF = 0.2 (4) with no improvement in the model.

## Supplementary Material

Crystal structure: contains datablock(s) global, I. DOI: 10.1107/S2056989021004813/ey2006sup1.cif

Structure factors: contains datablock(s) I. DOI: 10.1107/S2056989021004813/ey2006Isup2.hkl

Supporting information file. DOI: 10.1107/S2056989021004813/ey2006Isup3.cml

CCDC reference: 2082055

Additional supporting information:  crystallographic information; 3D view; checkCIF report

## Figures and Tables

**Figure 3:**
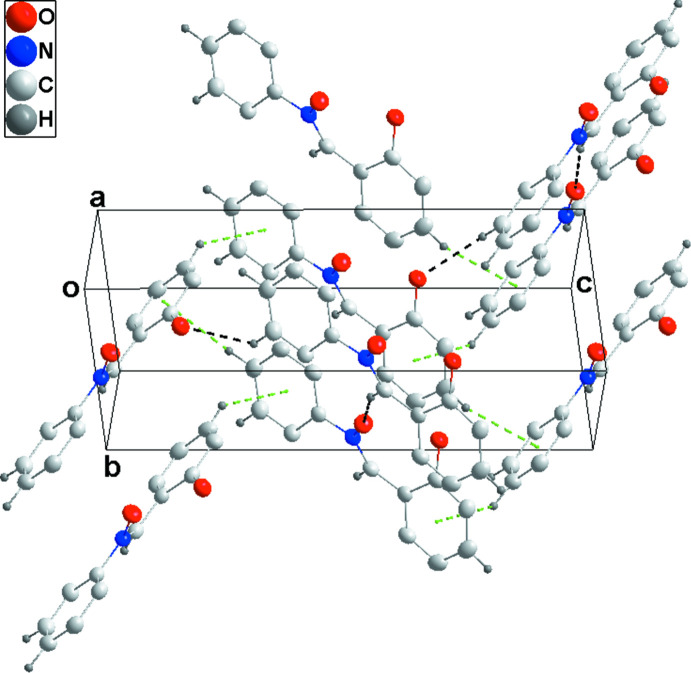
Packing viewed along the (120) direction with inter­molecular inter­actions shown as in Fig. 2 ▸.

**Table 1:** Hydrogen-bond geometry (Å, °) *Cg*1 and *Cg*2 are the centroids of the C1–C6 and C8–C13 aromatic rings, respectively.

*D*—H⋯*A*	*D*—H	H⋯*A*	*D*⋯*A*	*D*—H⋯*A*
O1—H1⋯O2	0.97	1.53	2.479 (2)	167
C7—H7⋯O2^i^	0.95	2.43	3.368 (3)	167
C10—H10⋯O1^ii^	0.95	2.53	3.227 (3)	131
C11—H11⋯*Cg*1^iii^	0.95	2.94	3.662 (3)	136
C4—H4⋯*Cg*2^iv^	0.95	2.77	3.545 (3)	140

Symmetry codes: (i) x-1, y, z; (ii) x-1, -y, z-{\script{1\over 2}}; (iii) x, -y, z-{\script{1\over 2}}; (iv) x, -y+1, z+{\script{1\over 2}}.

**Table 2:** Summary of short inter­atomic contacts (Å) in the title compound

Contact	Distance	Symmetry operation
O2⋯H7	2.43	1 + *x*, *y*, *z*
O1⋯H10	2.53	1 + *x*, −*y*, {1\over 2} + *z*
O2⋯H12	2.87	*x*, 1 + *y*, *z*
C3⋯H12	3.02	*x*, −*y*, {1\over 2} + *z*
H4⋯C11	2.86	*x*, 1 − *y*, {1\over 2} + *z*
H6⋯H13	2.46	−1 + *x*, 1 + *y*, *z*

**Table 3:** Percentage contributions of inter­atomic contacts to the Hirshfeld surface for the title compound

Contact	Percentage contribution
H⋯H	44.1
C⋯H/H⋯C	29.4
O⋯H/H⋯O	17.3
C⋯C	5.3
N⋯C/C⋯N	1.7
N⋯H/H⋯N	1.5
O⋯C/C⋯O	0.7

**Table 4:** Experimental details

Crystal data	
Chemical formula	C_13_H_11_NO_2_
*M*_r_	213.23
Crystal system, space group	Monoclinic, *P* *c*
Temperature (K)	150
*a*, *b*, *c* (Å)	5.5391 (1), 5.7873 (2), 16.0859 (4)
β (°)	99.067 (1)
*V* (Å^3^)	509.21 (2)
*Z*	2
Radiation type	Cu *K*α
μ (mm^−1^)	0.77
Crystal size (mm)	0.19 × 0.17 × 0.15

Data collection	
Diffractometer	Bruker D8 VENTURE PHOTON 100 CMOS
Absorption correction	Multi-scan (*SADABS*; Krause *et al.*, 2015 ▸)
*T*_min_, *T*_max_	0.77, 0.89
No. of measured, independent and observed [*I* > 2σ(*I*)] reflections	3578, 1654, 1607
*R*_int_	0.023
(sin θ/λ)_max_ (Å^−1^)	0.618

Refinement	
*R*[*F* ^2^ > 2σ(*F* ^2^)], *wR*(*F* ^2^), *S*	0.033, 0.084, 1.06
No. of reflections	1654
No. of parameters	145
No. of restraints	2
H-atom treatment	H-atom parameters constrained
Δρ_max_, Δρ_min_ (e Å^−3^)	0.18, −0.18
Absolute structure	Flack *x* determined using 611 quotients [(*I* ^+^)−(*I* ^−^)]/[(*I* ^+^)+(*I* ^−^)] (Parsons *et al.*, 2013 ▸).
Absolute structure parameter	0.30 (13)

Computer programs: *APEX3* and *SAINT* (Bruker, 2016 ▸), *SHELXT* (Sheldrick, 2015*a* ▸), *SHELXL* (Sheldrick, 2015*b* ▸), *ORTEP-3 for Windows* (Farrugia, 2012 ▸), *DIAMOND* (Brandenburg & Putz, 2012 ▸) and *PLATON* (Spek, 2020 ▸).

## supplementary crystallographic information

### Crystal data

**Table:**

C_13_H_11_NO_2_	*F*(000) = 224
*M**_r_* = 213.23	*D*_x_ = 1.391 Mg m^−^^3^
Monoclinic, *P**c*	Cu *K*α radiation, λ = 1.54178 Å
*a* = 5.5391 (1) Å	Cell parameters from 3430 reflections
*b* = 5.7873 (2) Å	θ = 5.6–72.4°
*c* = 16.0859 (4) Å	µ = 0.77 mm^−^^1^
β = 99.067 (1)°	*T* = 150 K
*V* = 509.21 (2) Å^3^	Block, yellow
*Z* = 2	0.19 × 0.17 × 0.15 mm

### Data collection

**Table:**

Bruker D8 VENTURE PHOTON 100 CMOS diffractometer	1654 independent reflections
Radiation source: INCOATEC IµS micro–focus source	1607 reflections with *I* > 2σ(*I*)
Mirror monochromator	*R*_int_ = 0.023
Detector resolution: 10.4167 pixels mm^-1^	θ_max_ = 72.4°, θ_min_ = 5.6°
ω scans	*h* = −6→6
Absorption correction: multi-scan (*SADABS*; Krause *et al.*, 2015)	*k* = −7→7
*T*_min_ = 0.77, *T*_max_ = 0.89	*l* = −19→19
3578 measured reflections	

### Refinement

**Table:**

Refinement on *F*^2^	Hydrogen site location: mixed
Least-squares matrix: full	H-atom parameters constrained
*R*[*F*^2^ > 2σ(*F*^2^)] = 0.033	*w* = 1/[σ^2^(*F*_o_^2^) + (0.0441*P*)^2^ + 0.084*P*] where *P* = (*F*_o_^2^ + 2*F*_c_^2^)/3
*wR*(*F*^2^) = 0.084	(Δ/σ)_max_ < 0.001
*S* = 1.06	Δρ_max_ = 0.18 e Å^−^^3^
1654 reflections	Δρ_min_ = −0.18 e Å^−^^3^
145 parameters	Absolute structure: Flack *x* determined using 611 quotients [(*I*^+^)-(*I*^-^)]/[(*I*^+^)+(*I*^-^)] (Parsons *et al.*, 2013).
2 restraints	Absolute structure parameter: 0.30 (13)

### Special details

**Table:**

Geometry. All esds (except the esd in the dihedral angle between two l.s. planes) are estimated using the full covariance matrix. The cell esds are taken into account individually in the estimation of esds in distances, angles and torsion angles; correlations between esds in cell parameters are only used when they are defined by crystal symmetry. An approximate (isotropic) treatment of cell esds is used for estimating esds involving l.s. planes.

### Fractional atomic coordinates and isotropic or equivalent isotropic displacement parameters (Å^2^)

**Table:**

	*x*	*y*	*z*	*U*_iso_*/*U*_eq_	
O1	0.8449 (3)	0.3532 (3)	0.64513 (12)	0.0342 (4)	
H1	0.841875	0.289527	0.589214	0.051*	
O2	0.7828 (3)	0.2215 (3)	0.49662 (12)	0.0338 (4)	
N1	0.5424 (3)	0.1864 (3)	0.48272 (13)	0.0267 (4)	
C1	0.4520 (4)	0.4953 (4)	0.57874 (14)	0.0260 (5)	
C2	0.6705 (4)	0.5169 (4)	0.63617 (15)	0.0280 (5)	
C3	0.7030 (4)	0.7093 (4)	0.68930 (16)	0.0328 (5)	
H3	0.852651	0.728354	0.726592	0.039*	
C4	0.5207 (5)	0.8717 (4)	0.68821 (18)	0.0348 (5)	
H4	0.545906	1.001606	0.724635	0.042*	
C5	0.3009 (4)	0.8470 (4)	0.63443 (17)	0.0349 (6)	
H5	0.174797	0.958261	0.634589	0.042*	
C6	0.2662 (4)	0.6611 (4)	0.58092 (16)	0.0304 (5)	
H6	0.114410	0.643651	0.544695	0.036*	
C7	0.3908 (4)	0.3082 (4)	0.51888 (15)	0.0270 (5)	
H7	0.222390	0.270734	0.504648	0.032*	
C8	0.4586 (4)	0.0034 (4)	0.42327 (14)	0.0263 (5)	
C9	0.2257 (4)	0.0070 (4)	0.37527 (15)	0.0304 (5)	
H9	0.116018	0.130115	0.380872	0.036*	
C10	0.1571 (4)	−0.1720 (4)	0.31930 (17)	0.0340 (5)	
H10	−0.001169	−0.171673	0.286384	0.041*	
C11	0.3176 (5)	−0.3521 (4)	0.31083 (16)	0.0327 (5)	
H11	0.269918	−0.473587	0.271973	0.039*	
C12	0.5480 (5)	−0.3533 (4)	0.35956 (18)	0.0324 (5)	
H12	0.657396	−0.477040	0.354374	0.039*	
C13	0.6199 (4)	−0.1748 (4)	0.41587 (16)	0.0301 (5)	
H13	0.778043	−0.175337	0.448865	0.036*	

### Atomic displacement parameters (Å^2^)

**Table:**

	*U*^11^	*U*^22^	*U*^33^	*U*^12^	*U*^13^	*U*^23^
O1	0.0260 (9)	0.0382 (9)	0.0363 (9)	0.0038 (6)	−0.0010 (7)	0.0001 (7)
O2	0.0137 (7)	0.0461 (9)	0.0418 (9)	−0.0015 (7)	0.0047 (7)	−0.0028 (8)
N1	0.0170 (9)	0.0340 (9)	0.0290 (10)	−0.0013 (7)	0.0031 (7)	0.0025 (8)
C1	0.0253 (12)	0.0287 (11)	0.0250 (12)	−0.0010 (8)	0.0073 (10)	0.0032 (8)
C2	0.0232 (12)	0.0314 (11)	0.0301 (12)	0.0004 (8)	0.0057 (10)	0.0066 (9)
C3	0.0308 (13)	0.0359 (12)	0.0320 (12)	−0.0082 (10)	0.0059 (10)	−0.0024 (10)
C4	0.0380 (14)	0.0300 (11)	0.0397 (13)	−0.0066 (9)	0.0166 (11)	−0.0022 (10)
C5	0.0346 (14)	0.0328 (12)	0.0403 (15)	0.0032 (9)	0.0150 (11)	0.0065 (10)
C6	0.0232 (11)	0.0362 (12)	0.0321 (12)	0.0026 (9)	0.0054 (9)	0.0071 (10)
C7	0.0185 (10)	0.0327 (11)	0.0293 (11)	0.0009 (8)	0.0028 (9)	0.0049 (9)
C8	0.0241 (11)	0.0292 (11)	0.0258 (13)	−0.0022 (8)	0.0044 (10)	0.0029 (8)
C9	0.0218 (11)	0.0379 (12)	0.0316 (13)	0.0017 (9)	0.0053 (10)	−0.0012 (9)
C10	0.0246 (11)	0.0448 (13)	0.0329 (12)	−0.0037 (9)	0.0052 (10)	−0.0013 (11)
C11	0.0328 (12)	0.0340 (12)	0.0328 (13)	−0.0066 (9)	0.0096 (10)	−0.0021 (10)
C12	0.0336 (12)	0.0307 (11)	0.0341 (12)	0.0044 (9)	0.0096 (10)	0.0023 (10)
C13	0.0249 (11)	0.0350 (12)	0.0307 (13)	0.0025 (8)	0.0056 (9)	0.0053 (9)

### Geometric parameters (Å, º)

**Table:**

O1—C2	1.345 (3)	C5—H5	0.9500
O1—H1	0.9697	C6—H6	0.9500
O2—N1	1.331 (2)	C7—H7	0.9500
N1—C7	1.302 (3)	C8—C13	1.382 (3)
N1—C8	1.454 (3)	C8—C9	1.395 (3)
C1—C2	1.407 (3)	C9—C10	1.386 (4)
C1—C6	1.411 (3)	C9—H9	0.9500
C1—C7	1.453 (3)	C10—C11	1.391 (4)
C2—C3	1.398 (4)	C10—H10	0.9500
C3—C4	1.377 (4)	C11—C12	1.389 (4)
C3—H3	0.9500	C11—H11	0.9500
C4—C5	1.385 (4)	C12—C13	1.390 (4)
C4—H4	0.9500	C12—H12	0.9500
C5—C6	1.372 (4)	C13—H13	0.9500
			
C2—O1—H1	105.1	N1—C7—C1	126.9 (2)
C7—N1—O2	122.78 (18)	N1—C7—H7	116.6
C7—N1—C8	121.79 (18)	C1—C7—H7	116.6
O2—N1—C8	115.43 (17)	C13—C8—C9	121.2 (2)
C2—C1—C6	118.6 (2)	C13—C8—N1	117.2 (2)
C2—C1—C7	125.97 (19)	C9—C8—N1	121.66 (19)
C6—C1—C7	115.4 (2)	C10—C9—C8	118.9 (2)
O1—C2—C3	118.3 (2)	C10—C9—H9	120.5
O1—C2—C1	122.5 (2)	C8—C9—H9	120.5
C3—C2—C1	119.1 (2)	C9—C10—C11	120.6 (2)
C4—C3—C2	120.8 (2)	C9—C10—H10	119.7
C4—C3—H3	119.6	C11—C10—H10	119.7
C2—C3—H3	119.6	C12—C11—C10	119.6 (2)
C3—C4—C5	120.5 (2)	C12—C11—H11	120.2
C3—C4—H4	119.7	C10—C11—H11	120.2
C5—C4—H4	119.7	C11—C12—C13	120.5 (2)
C6—C5—C4	119.6 (2)	C11—C12—H12	119.8
C6—C5—H5	120.2	C13—C12—H12	119.8
C4—C5—H5	120.2	C8—C13—C12	119.2 (2)
C5—C6—C1	121.2 (2)	C8—C13—H13	120.4
C5—C6—H6	119.4	C12—C13—H13	120.4
C1—C6—H6	119.4		
			
C6—C1—C2—O1	172.1 (2)	C6—C1—C7—N1	153.4 (2)
C7—C1—C2—O1	−4.2 (3)	C7—N1—C8—C13	−153.0 (2)
C6—C1—C2—C3	−4.4 (3)	O2—N1—C8—C13	27.7 (3)
C7—C1—C2—C3	179.3 (2)	C7—N1—C8—C9	27.3 (3)
O1—C2—C3—C4	−174.0 (2)	O2—N1—C8—C9	−152.0 (2)
C1—C2—C3—C4	2.6 (3)	C13—C8—C9—C10	−0.1 (3)
C2—C3—C4—C5	0.1 (4)	N1—C8—C9—C10	179.6 (2)
C3—C4—C5—C6	−1.0 (4)	C8—C9—C10—C11	−0.2 (4)
C4—C5—C6—C1	−0.9 (4)	C9—C10—C11—C12	0.6 (4)
C2—C1—C6—C5	3.6 (3)	C10—C11—C12—C13	−0.7 (4)
C7—C1—C6—C5	−179.7 (2)	C9—C8—C13—C12	0.0 (3)
O2—N1—C7—C1	−0.4 (3)	N1—C8—C13—C12	−179.8 (2)
C8—N1—C7—C1	−179.7 (2)	C11—C12—C13—C8	0.4 (4)
C2—C1—C7—N1	−30.2 (3)		

### Hydrogen-bond geometry (Å, º)

*Cg*1 and *Cg*2 are the centroids of the C1–C6 and C8–C13 aromatic rings, respectively.

**Table:**

*D*—H···*A*	*D*—H	H···*A*	*D*···*A*	*D*—H···*A*
O1—H1···O2	0.97	1.53	2.479 (2)	167
C7—H7···O2^i^	0.95	2.43	3.368 (3)	167
C10—H10···O1^ii^	0.95	2.53	3.227 (3)	131
C11—H11···*Cg*1^iii^	0.95	2.94	3.662 (3)	136
C4—H4···*Cg*2^iv^	0.95	2.77	3.545 (3)	140

Symmetry codes: (i) *x*−1, *y*, *z*; (ii) *x*−1, −*y*, *z*−1/2; (iii) *x*, −*y*, *z*−1/2; (iv) *x*, −*y*+1, *z*+1/2.
